# Characterization of the Complete Mitochondrial Genome of Dwarf Form of Purpleback Flying Squid (*Sthenoteuthis oualaniensis*) and Phylogenetic Analysis of the Family Ommastrephidae

**DOI:** 10.3390/genes16020226

**Published:** 2025-02-15

**Authors:** Wenjuan Duo, Lei Xu, Mohd Johari Mohd Yusof, Yingmin Wang, Seng Beng Ng, Feiyan Du

**Affiliations:** 1 Faculty of Design and Architecture, Universiti Putra Malaysia, Serdang 43400, Selangor, Malaysia; simonaduo5461@gmail.com (W.D.); m_johari@upm.edu.my (M.J.M.Y.); 2 South China Sea Fisheries Research Institute, Chinese Academy of Fishery Sciences, Guangzhou 510300, China; xulei@scsfri.ac.cn; 3 Sanya Tropical Fisheries Research Institute, Sanya 572018, China; 4 School of Environmental Sciences, James Cook University, Singapore 387380, Singapore; 5 Department of Multimedia, Universiti Putra Malaysia, Serdang 43400, Selangor, Malaysia

**Keywords:** Ommastrephidae, mitochondrial genome, phylogenetic, *Sthenoteuthis*, dwarf form

## Abstract

Background: The Ommastrephidae family of cephalopods is important in marine ecosystems as both predators and prey. Species such as *Todarodes pacificus*, *Illex argentinus*, and *Dosidicus gigas* are economically valuable but are threatened by overfishing and environmental changes. The genus *Sthenoteuthis*, especially *S. oualaniensis*, shows significant morphological and genetic variation, including medium-sized and dwarf forms found in the South China Sea. Methods: Specimens of *S. oualaniensis* were collected from the South China Sea, their genomic DNA sequenced, and phylogenetic relationships analyzed using mitochondrial genomes from various Ommastrephidae species. Results: The study presents the complete mitochondrial genome of the dwarf form of *S. oualaniensis* (20,320 bp) and compares it with the medium-sized form, revealing a typical vertebrate structure with 13 protein-coding genes, 21 tRNA genes, and 2 rRNA genes, along with a strong AT bias. Nucleotide composition analysis shows a 12% genetic divergence between the two forms, suggesting a recent common ancestor and potential cryptic speciation, with all protein-coding genes exhibiting purifying selection based on Ka/Ks ratios below 1. Conclusions: The mitochondrial genome of the dwarf form of *S. oualaniensis* shows a close evolutionary relationship with the medium-sized form and a 12% genetic divergence, suggesting potential cryptic speciation. These findings underscore the importance of mitochondrial analysis in understanding speciation and guiding future conservation efforts.

## 1. Introduction

The family Ommastrephidae, a prominent group of cephalopods, plays a crucial ecological role and possesses significant biological value in marine ecosystems. Widely distributed across tropical, subtropical, and certain temperate waters, ommastrephid squids occupy surface to midwater zones, acting as vital conduits for energy transfer within marine food webs. Their rapid growth rates, high reproductive potential, and remarkable adaptability to environmental changes underpin their ecological importance and contribute to the dynamic balance of marine ecosystems [1,2,3]. As mid-to-high trophic level predators, they feed on small fish, crustaceans, and other cephalopods, while simultaneously serving as key prey for numerous fish species, marine mammals, and seabirds. This dual role positions them as integral components of marine food webs, playing a critical role in sustaining ecosystem dynamics and ensuring ecological stability [4,5,6]. Species of the family Ommastrephidae are considered ideal models for studying ecological adaptation, population dynamics, and evolutionary mechanisms, owing to their complex population structures, remarkable morphological diversity, and rapid life cycles [7,8]. Their heightened sensitivity to environmental fluctuations further underscores their value as indicator species for assessing the health of marine ecosystems and monitoring the effects of climate change [9]. These research advancements not only enhance our understanding of biodiversity within the family but also offer critical theoretical support for the development of evidence-based fisheries management strategies [10,11]. Additionally, the family Ommastrephidae, as a highly valuable marine biological resource, occupies a critical position in global fisheries. Representative species such as the jumbo squid (*D. gigas*), the Argentinean short-finned squid (*I. argentinus*), and the Japanese common squid (*T. pacificus*) are among the primary targets in global cephalopod fisheries. These species are distinguished by their broad distribution, high yields, and the use of advanced fishing techniques, making them essential resources for both coastal and distant-water fisheries. However, the exploitation of Ommastrephidae resources is subject to considerable spatiotemporal variability, with factors such as population size, distribution range, and resource abundance being influenced by both environmental fluctuations and human fishing activities [12,13,14]. In recent years, the development of Ommastrephidae fisheries has become increasingly globalized, with a notable expansion in fishing grounds such as the Northwest Pacific, South Atlantic, and Eastern Pacific Oceans. Species within this family exhibit rapid growth, high reproductive rates, and short life cycles, enabling them to withstand certain fishing pressures [15,16,17]. Nevertheless, their sensitivity to environmental fluctuations, including marine surface temperature (MST), oceanic currents, and basal productivity, often leads to substantial variability in resource abundance. For instance, climatic phenomena such as El Niño and La Niña significantly influence the distribution and yield of *D. gigas* [18,19,20]. Additionally, overfishing presents a considerable threat to certain populations, particularly in unregulated high-seas regions. These challenges underscore the urgent need for comprehensive scientific assessments and the adoption of robust management approaches to safeguard the long-term viability of these crucial fishery resources [12,21,22].

The family Ommastrephidae comprises five subfamilies (Ommastrephinae, Ornithoteuthinae, Todarodinae, Todaropsinae, and Illicinae) and includes several genera such as *Todarodes*, *Illex*, *Dosidicus*, *Nototodarus*, and *Sthenoteuthis*. Each genus contains species uniquely adapted to specific ecological niches [23,24,25,26]. For instance, the genus *Todarodes* includes economically significant species like *T. pacificus*, while *Illex* encompasses highly migratory species such as *I. argentinus* and *I. illecebrosus*. Among the members of *Dosidicus*, *D. gigas*, commonly known as the jumbo squid, is particularly noteworthy as one of the largest and most ecologically influential species, exerting a dominant role in the Eastern Pacific ecosystem [2,27].

*S. oualaniensis* Lesson 1830 from the genus *Sthenoteuthis*, commonly known as the purpleback flying squid, belonging to the family Ommastrephidae, is extensively found in the equatorial and subtropical areas of both the Pacific and Indian Oceans [28]. Its range extends from 30° N to 25° S, with occasional occurrences as far north as 40° N and south to 47° S, and recent reports have documented its presence as far north as 60° N. While it is absent from temperate Pacific waters, the species is most prevalent in the South China Sea and the northwestern part of the Indian Ocean [2,28,29]. Similar to other species in the Ommastrephidae family, *S. oualaniensis* displays several forms that differ in mature size and the presence of a prominent large dorsal photophore. This species includes two major morphs in the South China Sea: a dwarf form and a medium-sized form. The dwarf form, which does not possess a dorsal photophore, exhibits a mantle length between 9 and 12 cm. In contrast, the medium-sized form, distinguished by the presence of a photophore, features females maturing at a mantle length of 15 to 40 cm, while males mature at a length of 12 to 24 cm. The current taxonomic classification of *S. oualaniensis* is complicated by the limited understanding of its life cycles, foraging behaviors, and reproductive patterns, leading to ongoing debates regarding its classification [1,30]. Previous molecular analyses of *S. oualaniensis* revealed four genetically distinct lineages in the Indian Ocean, highlighting significant phylogeographic structuring and uncovering five evolutionary units, with evidence suggesting sympatric speciation of the dwarf form [31]. Xu et al. [32] investigated the genetic and morphological variation of *S. oualaniensis* in the South China Sea, identifying two distinct lineages: a dwarf form confined to equatorial waters and a medium-sized form with a broader distribution. These results suggest a pseudo-cryptic species complex, supported by both genetic markers and morphology, with the medium-sized form further subdivided into two clades. However, these studies are limited by the small number of species analyzed and the use of short sequence fragments, underscoring the necessity for further research incorporating a larger species sample and more comprehensive sequence data to achieve more robust and reliable phylogenetic insights. Mitochondrial DNA (mtDNA) is widely used in phylogenetic studies due to its relatively high mutation rate, absence of recombination, and maternal inheritance, which make it a useful tool for tracing evolutionary relationships and population structure [33,34]. However, it is essential to acknowledge the limitations of mtDNA. Since it is inherited maternally, it does not capture paternal genetic contributions, which could limit our understanding of the full genetic diversity and evolutionary history of species. Furthermore, the lack of recombination in mtDNA results in a more static genetic marker, potentially overlooking the finer-scale genetic variation that could be present in more dynamic genomic regions [35,36]. In this study, we conducted the sequencing, assembly, and annotation of the complete mitochondrial genome for the dwarf form of *S. oualaniensis* and compared it with that of the medium-sized form for the first time. Additionally, we analyzed the phylogenetic relationships among 7 out of the 23 recognized species within the family Ommastrephidae using complete mitochondrial genomes. Specifically, our objective is to elucidate the phylogenetic position of *S. oualaniensis* and to investigate the phylogenetic relationships between species across different subfamilies within the Ommastrephidae family.

## 2. Materials and Methods

### 2.1. Sample Collection and Identification of Morphology

Specimens of *S. oualaniensis* were obtained from the South China Sea (11°26′ N, 114°01′ E) on 11 April 2017, using a light falling net deployed by the commercial fishing vessel “Guibeiyu 96886”. The collected samples were immediately stored in a freezer at −20 °C after morphological identification of the genus *Sthenoteuthis*. This study adhered to the guidelines established by the International Union for Conservation of Nature (IUCN) for studies involving endangered species, along with the rules set forth by the Convention on Biological Diversity (CBD) and the Convention on International Trade in Endangered Species of Wild Fauna and Flora (CITES). *S. oualaniensis* was classified as “Least Concern” on the IUCN Red List of Threatened Species [37]. The different forms were identified at the Guangdong Provincial Key Laboratory of Fishery Ecology and Environment based on the following morphological characteristics. Mantle Length (ML): The medium-sized form exhibits significantly greater body size compared to the dwarf form, with mantle lengths ranging from 120 mm to 400 mm, whereas the dwarf form typically measures between 90 mm and 120 mm. Arm Length (AL): Distinct differences in arm length are evident, particularly in the first pair of arms (AL1), which serve as a critical morphological feature for distinguishing the two forms. Tentacle Length (TL): Tentacle length also varies, with the dwarf form having noticeably shorter tentacles compared to the medium-sized form. Fin Length (FL) and Fin Width (FW): The size and shape of fins differ between the forms, offering another distinguishing morphological characteristic. Gonad Maturity: The medium-sized form typically exhibits delayed gonadal maturation, while the dwarf form tends to mature earlier, highlighting a significant reproductive difference. Photophore Presence: Photophore development is a key differentiator. The medium-sized form usually possesses photophores on its dorsal side, while individuals from the dwarf form lack photophores or exhibit degenerated ones [2,30,38]. The specimens in this study had a small mantle length (ML) of only 10 cm, tentacle lengths of 4 cm, lacked photophores on the dorsal side, and exhibited mature gonads.

### 2.2. DNA Extraction, Sequencing and Annotation

Genomic DNA was isolated from the body muscle tissue of a dwarf form of *S. oualaniensis* specimen using the TIANamp Marine Animals DNA Kit (TIANGEN, Beijing, China), in accordance with the manufacturer’s protocol specifically designed for extracting DNA from marine animal muscle tissue. The specimen and the extracted DNA have been stored at the Guangdong Provincial Key Laboratory of Fishery Ecology and Environment, registered under the voucher number SCS2017-S10-450. Genomic DNA samples, characterized by an A260/A280 ratio between 1.8 and 2.1, were utilized as templates for PCR amplification. DNA sequencing was conducted using an ABI 3730xl DNA automatic sequencer with genomic DNA as the template. The PCR primers were developed based on highly conserved regions of tRNA sequences from related species. Additionally, species-specific primers were designed as required, drawing from previously acquired sequence data [39] (Table S1). PCR amplification was carried out using TopTaq DNA polymerase (Qiagen, Hilden, Germany), following the manufacturer’s protocol with annealing temperatures varying between 45 °C and 55 °C. Sanger sequencing was conducted on the PCR products in both directions using the primer walking method to ensure comprehensive and accurate sequence coverage. The COI sequence from the dwarf form of *S. oualaniensis* served as the reference seed for iterative assembly using MITObim version 1.8 [40]. The mitogenome assembly and annotation were performed using SeqMan version 7.1.0, followed by manual verification [41]. Transfer RNA genes were predicted using tRNAScan-SE 1.21 via its online platform, utilizing the default search parameters and selecting “Mito/chloroplast” as the genetic source [42]. The mitochondrial genome of the species was mapped using assembly and annotation data. A diagram was generated through the GCView Server to effectively visualize the genomic information [43]. Nucleotide composition skewness was determined using the following equations: $A/T-skew=\frac{A-T}{A+T}$ and $G/C-skew=\frac{G-C}{G+C}$ [44]. The relative synonymous codon usage (RSCU) for the 13 protein-coding genes (PCGs) was calculated using MEGA software, version 6.0 [45]. This analysis provides insights into codon preference, which is essential for understanding genetic coding patterns and evolutionary adaptations within the mitochondrial genome. The mitochondrial genome of the dwarf form of *S. oualaniensis* was compared to that of the medium-sized form of *S. oualaniensis*, as documented in our previous study (MT661575) [46]. This comparative analysis aimed to identify differences and similarities in genomic features between the two forms, providing insights into their evolutionary relationship and potential species differentiation. Additionally, non-synonymous (Ka) and synonymous (Ks) substitution rates were calculated using DnaSP v6, offering valuable insights into the evolutionary dynamics of the mitochondrial genome [47].

### 2.3. Phylogenetic Analysis

A total of eight mitochondrial genomes from the Ommastrephidae family, retrieved from the NCBI database, were analyzed to investigate phylogenetic relationships, with *Architeuthis dux* (KC701744) used as the outgroup (Table S2). *A. dux* was selected as the outgroup due to its well-characterized mitochondrial genome and its phylogenetic position within Oegopsida, making it an appropriate reference for rooting the tree. Other deep-sea cephalopods were not included primarily due to the limited availability of high-quality, complete mitochondrial genomes in public databases. The sequences underwent preliminary processing for visualization and assembly within BioEdit, after which they were automatically aligned utilizing the default settings. To ensure the robustness of our alignment, gaps and missing data were carefully treated [48].

Phylogenetic analyses were conducted using PhyloSuite v1.2.1 [49] through both maximum likelihood (ML) and Bayesian inference (BI) methods. ModelFinder was used to select the best-fit partition model (edge-unlinked) using BIC criterion [50]. Maximum likelihood (ML) phylogenies were constructed using IQ-TREE [51] with the edge-linked partition model, employing 5000 ultrafast bootstrap replicates and the Shimodaira–Hasegawa-like approximate likelihood ratio test [52] to assess branch support. Bayesian inference (BI) phylogenies were generated using MrBayes 3.2.6 [53] under the partition model (2 parallel runs, 7,711,800 generations). The BI analysis consisted of two parallel runs over 10,000 generations, with the initial 25% of sampled data discarded as burn-in to ensure convergence and reliability.

## 3. Results

### 3.1. Mitogenome Structure

**Figure 1:**
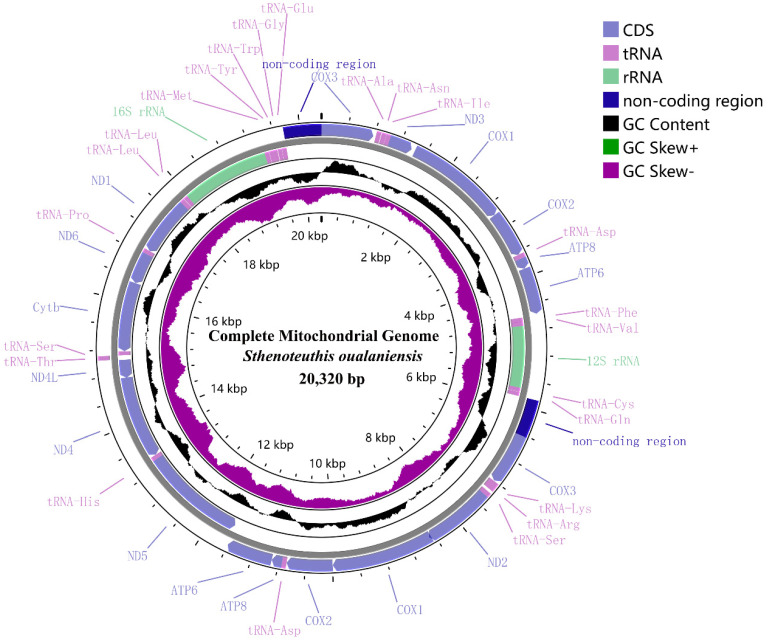
Annotated mitochondrial genome of dwarf form of *S. oualaniensis*. Blue bars denote protein-coding genes, virescent bars represent rRNA genes, and lavender bars indicate tRNA genes. The direction of transcription is shown by the orientation of gene arrows: arrows pointing to the right indicate the heavy strand, while those pointing to the left denote the light strand. The black circle represents GC content, with outward projections indicating GC content above the average level and inward projections indicating below-average content. The GC skew is depicted using purple and green circles, where green represents negative GC skew and deep purple indicates positive GC skew.

The mitochondrial genome of the dwarf form of *S. oualaniensis* is a closed circular molecule measuring 20,320 bp (GenBank accession number MW542205). The mitochondrial genome exhibits an overall base composition of A (37.23%), T (32.78%), G (10.53%), and C (19.46%), resulting in a pronounced AT bias of 70.01%. Its structure is highly conserved, adhering to the typical vertebrate mitochondrial genome organization. The genome comprises 2 rRNA genes (*12S rRNA* and *16S rRNA*), 21 tRNA genes, and 13 protein-coding genes (PCGs). These PCGs encompass the cytochrome b gene (*Cytb*), two ATP synthase subunits (*ATP6*, *ATP8*), three cytochrome c oxidase subunits (*COX1*, *COX2*, *COX3*), and seven NADH dehydrogenase subunits (*ND1*, *ND2*, *ND3*, *ND4*, *ND4L*, *ND5*, *ND6*). The light strand (L-strand) of the mitochondrial genome encodes thirteen transfer RNA genes (tRNA-Met, tRNA-Tyr, tRNA-Trp, tRNA-Gly, tRNA-Glu, tRNA-Phe, tRNA-Val, tRNA-Cys, tRNA-Gln, tRNA-His, tRNA-Ser, tRNA-Pro, and tRNA-Leu), six protein-coding genes (*ND1*, *ND4*, *ND4L*, *ND5*, *ND6*, and *Cytb*), and two rRNA genes (*12S rRNA* and *16S rRNA*). Conversely, the remaining genes are encoded on the heavy strand (H-strand) of the mitochondrial genome (Figure 1). The ribosomal RNA genes have lengths of 1425 bp for the *16S rRNA* and 990 bp for the *12S rRNA*, respectively. All 21 tRNA genes display a full cloverleaf secondary structure, with their lengths varying between 65 and 74 bp. The mitochondrial genome additionally contains seven duplicated genes (*ATP6*, *ATP8*, *COX1*, *COX2*, *COX3*, tRNA-Leu, and tRNA-Asp), a feature that is characteristic of Oegopsid cephalopod mitogenomes [39]. The mitochondrial genome of the dwarf form of *S. oualaniensis* demonstrated a positive A/T skew of 0.0636 and a negative G/C skew of −0.2978, indicating a higher proportion of cytosine compared to guanine.

The protein-coding genes in the mitochondrial genome of the dwarf form of *S. oualaniensis* collectively cover a total length of 15,041 bp, representing 74.02% of the entire mitochondrial genome. This region displayed a significant AT bias, with A + T content comprising 68.32%, and collectively encoded 4788 amino acid residues. Within the set of protein-coding genes, *ND1*, *ND4*, *ND4L*, *ND5*, *ND6*, and *Cytb* were positioned on the light strand (L-strand), whereas the other genes were located on the heavy strand (H-strand). Of these, the *ND5* gene was the longest, spanning 1698 bp and encoding 565 amino acids, while the ATP8 gene was the shortest, at only 156 bp, encoding 51 amino acids. In the mitochondrial genome of the dwarf form of *S. oualaniensis*, the majority of protein-coding genes (PCGs) employ the initiation codon ATG, with the exception of *ND4* and *Cytb*, which utilize *ATA*. All thirteen PCGs have complete termination codons: seven genes (*COX3*, *ND3*, *COX1*, *ATP8*, *ND2*, *ND1*, and *ND5*) terminate with TAA, five genes (*ATP6*, *ND4L*, *ND6*, *Cytb*, and *ND4*) with TAG, and one gene (*COX2*) with AAT. In comparison, the mitochondrial genome of the medium-sized form of *S. oualaniensis* shows some differences in codon usage. The initiation codon ATG is used by most PCGs, except for *ND1* (which uses ATA) and *ND5* and *ND2* (both of which use ATT). Like the dwarf form, all 13 PCGs in the medium-sized form possess complete termination codons. Six genes (*ND2*, *ND3*, *ND5*, *COX2*, *COX3*, and *ATP8*) terminate with TAA, while seven genes (*Cytb*, *COX1*, *ND1*, *ND4*, *ND4L*, *ND6*, and *ATP6*) use TAG as their termination codon (Table 1).

**Figure 2:**
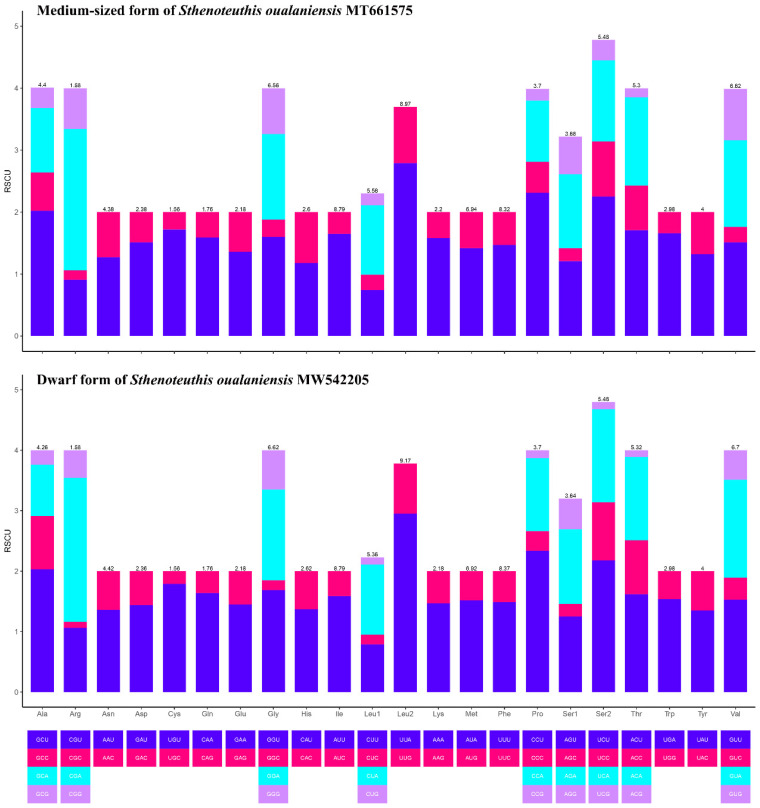
Relative synonymous codon usage (RSCU) patterns in the medium-sized and dwarf forms of *S. oualaniensis*.

**Figure 3:**
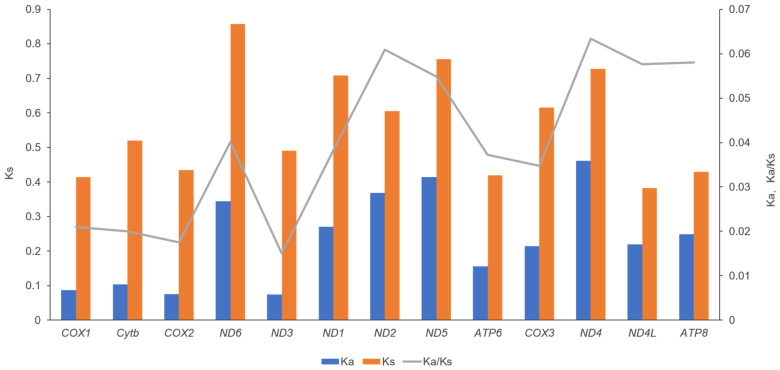
The ratio of nonsynonymous to synonymous substitutions (Ka/Ks) across 13 protein coding genes in two forms of *S. oualaniensis*.

To investigate codon usage patterns and amino acid distribution, the amino acid composition and relative synonymous codon usage (RSCU) of the 13 protein-coding genes were analyzed in the mitochondrial genomes of both the dwarf and medium-sized forms of *S. oualaniensis*. This comparative analysis provides insights into codon preference and the functional implications of amino acid composition in these two forms. The RSCU analysis revealed that the mitochondrial genomes of both forms of *S. oualaniensis* exhibited the highest frequency for codons corresponding to the amino acids Leu, Ile, Phe, Met, Val, and Gly, while codons for Cys were less frequent (Figure 2). Two identical long non-coding regions (LNCRs), measuring 561 bp and 562 bp, respectively, were identified on the heavy (H) strand of the mitochondrial genomes in both the medium-sized and dwarf forms of *S. oualaniensis*. The first long non-coding RNA (LNCR) is situated in the region between the tRNA-Gln gene and the *COX3* gene, whereas the second LNCR is found in the interval between the tRNA-Glu gene and the duplicate copy of the *COX3* gene. These non-coding regions likely play critical roles in mitochondrial gene regulation, including DNA replication and transcription. The ratio of non-synonymous to synonymous substitution rates (Ka/Ks) was employed to evaluate the selective pressure acting on 13 protein-coding genes (PCGs) across the two forms of *S. oualaniensis*. The Ka/Ks ratios for all PCGs remained consistently below 1 (Figure 3), indicating strong purifying selection acting on these genes in both forms. Among the PCGs, *ND4* exhibited the highest Ka/Ks value, suggesting a relatively faster evolutionary rate compared to other mitochondrial genes. In contrast, *COX2* showed the lowest Ka/Ks values, highlighting significant selective constraints and slower evolutionary rates for these genes.

### 3.2. Phylogenetic Tree

**Figure 4:**
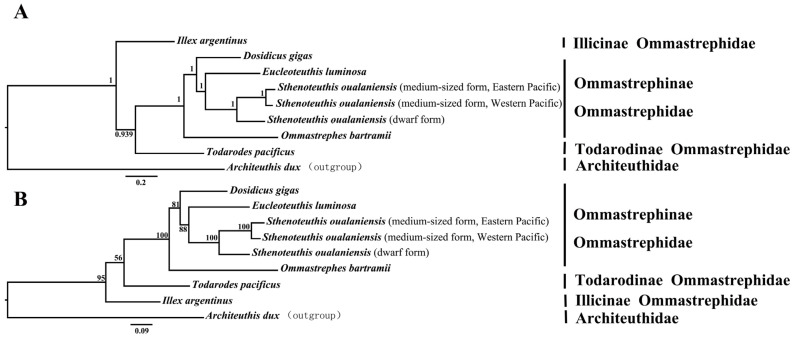
Bayesian inference (BI) (**A**) and maximum likelihood (ML) (**B**) phylogenetic trees illustrating the evolutionary relationships among cephalopod species based on mitochondrial genome sequences. The tree is rooted with *A. dux* as the outgroup. Posterior probabilities and support value are displayed at the nodes. Notable clades include multiple mitochondrial haplotypes of *S. oualaniensis* forming a well-supported cluster, and a close relationship between *D. gigas* and *Eucleoteuthis luminosa*. The longer branch lengths of *T. pacificus* and *I. argentinus* indicate greater genetic divergence compared to other taxa. The scale bar represents genetic distance.

To ascertain the phylogenetic position of the dwarf form of *S. oualaniensis*, all available mitochondrial genomes from the family Ommastrephidae were retrieved from the NCBI database. This dataset included the mitochondrial genome of the medium-sized form of *S. oualaniensis* from our previous study [46]. Additionally, the mitochondrial genome of Architeuthis dux was used as an outgroup. Phylogenetic trees were generated using Maximum Likelihood (ML) and Bayesian Inference (BI) approaches, based on the nucleotide sequences from 13 protein-coding genes (PCGs). The phylogenetic analyses revealed congruent topological structures between the Maximum Likelihood (ML) and Bayesian Inference (BI) methods, strongly supporting the delineation of several clades based on the available data (Figure 4). The main clade consists of species from the genera *Dosidicus*, *Eucleoteuthis*, *Sthenoteuthis*, and *Ommastrephes*, all members of the subfamily Ommastrephinae. In contrast, the remaining clades include species from the genera *Illex* and *Todarodes*, which belong to the subfamilies Illicinae and Todaropsinae, respectively. The phylogenetic analyses revealed that the target species, the dwarf form of *S. oualaniensis*, and the medium-sized form of *S. oualaniensis* are clustered together as a monophyletic group. This clustering indicates that the two forms share a recent common ancestor and exhibit a closer relationship to each other compared to any other species within the Ommastrephidae family. All these results underscore the reliability of the available data in elucidating the evolutionary relationships among species within this family.

## 4. Discussion

The mitochondrial DNA of Metazoa serves as an excellent model for investigating evolutionary genomics. With the rapid advancements in DNA sequencing technologies and bioinformatics, mitochondrial genomes of marine organisms have increasingly been utilized in fields such as species identification and phylogenetic research [54,55,56,57,58,59,60,61]. This research provided the full mitochondrial genome sequence of the dwarf form of *S. oualaniensis*, comprising a total length of 20,320 bp. The structural features of this genome closely resemble those of other Oegopsida species, demonstrating a significant level of conservation within this order. Additionally, the gene lengths observed in *S. oualaniensis* are largely consistent with those of previously published Oegopsida mitochondrial genomes, further supporting the notion of extensive conservation across species within this group. These findings highlight the structural stability of mitochondrial genomes in Oegopsida and suggest that the essential functions of these genomes are maintained across species [25,62,63,64].

AT-skew and GC-skew values provide crucial insights into the nucleotide composition bias in DNA sequences, particularly within mitochondrial genomes. These values reflect the relative abundance of adenine (A) versus thymine (T) (AT-skew) and guanine (G) versus cytosine (C) (GC-skew) within a genome. They are frequently utilized to assess the asymmetry between the light (L-strand) and heavy (H-strand) strands of mitochondrial DNA. The AT-skew value primarily reflects the asymmetric distribution of A and T, which is often associated with the directionality of transcription and the replication mechanisms within the genome. Conversely, the GC-skew value highlights the asymmetry between C and G, typically correlating with replication origins and the characteristics of the lagging strand [44]. The mitochondrial DNA sequence of the dwarf form of *S. oualaniensis* exhibits a high AT content, demonstrating a clear AT-bias, a feature also observed in other cephalopod species. In cephalopods, AT/GC-skew values show considerable variation across different species, reflecting distinct mitochondrial genome characteristics. Generally, AT-skew values in cephalopods are positive, indicating an overrepresentation of adenine (A) relative to thymine (T) on the heavy strand. This pattern is consistent with typical replication and transcription mechanisms observed in mitochondrial genomes. In contrast, GC-skew values in cephalopods tend to be negative, indicating a greater proportion of cytosine (C) relative to guanine (G) on the heavy strand. These base preferences may arise from factors such as natural mutations and selective pressures acting during the processes of replication and transcription [25,65,66,67].

The non-coding regions (NCRs) within the mitochondrial genome play a primary role in regulating gene expression and are essential for the replication and transcription of mitochondrial DNA. In the mitochondrial genome of the dwarf form of *S. oualaniensis*, the non-coding regions are similar to those found in other Oegopsida species, consisting mainly of two copies of long non-coding regions (LNCRs) located on the heavy (H) strand. NCRs harbor critical elements for initiating the replication and transcription of mitochondrial DNA. These regions contain promoters for RNA polymerase and sequences that bind replication proteins, ensuring proper mitochondrial genome maintenance and gene expression. NCRs also are hotspots for genetic recombination and rearrangement, which may drive mitochondrial genome evolution in cephalopods. Tomita, Yokobori, Oshima, Ueda, and Watanabe [65] reported that the mitochondrial genome of *Loligo bleekeri* contains 19 noncoding regions, three of which (515, 507, and 509 bp) are nearly identical, suggesting they originated from duplication events in an ancestral genome. The study further proposed that the dispersion of tRNA genes in *Loligo* is associated with the multiplication of noncoding regions, highlighting a potential mechanism underlying mitochondrial genome evolution in cephalopods. In addition, these non-coding regions represent some of the fastest-evolving sequences in mitochondrial DNA, playing a significant role in the molecular evolution of cephalopods. Due to their high evolutionary rate, these regions are widely utilized in studies of cephalopod population genetics and molecular systematics [68,69]. The mitochondrial genome of the dwarf form of *S. oualaniensis* exhibits seven gene duplications, including *ATP6*, *ATP8*, *COX1*, *COX2*, *COX3*, tRNA-Leu, and tRNA-Asp. These duplications are characteristic features of Oegopsid cephalopod mitogenomes [39]. Duplicated genes in cephalopods may arise through mechanisms such as gene duplication, genome rearrangement, or errors in mitochondrial DNA replication, processes potentially linked to their complex life histories and adaptation to environmental pressures. The emergence of duplicated genes holds significant evolutionary importance for cephalopods. On one hand, it provides raw material for gene functional diversification, potentially facilitating the development of new functions to adapt to diverse ecological niches. On the other hand, duplicated genes may enhance the flexibility of the mitochondrial genome, contributing to optimized energy metabolism and adaptation to varying environmental conditions, thereby improving the competitive survival capabilities of cephalopods [70,71]. Later studies have suggested that the mitochondrial genomes of Sepiida and Oegopsida likely evolved through a process of whole-genome duplication followed by random gene loss, originating from the mitochondrial genome of Octopoda [24,72]. In the mitochondrial genome of the dwarf form of *S. oualaniensis* analyzed in this study, the duplicated gene copies were found to be highly conserved. Among them, only the *COX2* gene exhibited five synonymous mutation sites, yet the encoded amino acid sequences remained identical. This observation suggests that the duplicated gene copies retain identical functions. Kawashima et al. [72] reported that in the mitochondrial genome of *Bathyteuthis*, a single amino acid change from A to V was observed in the duplicated *COX1* genes. In contrast, two amino acid substitutions—W to Y and N to D—were identified in the duplicated *COX2* genes. Notably, the second copy of the *COX3* gene displayed multiple deletions and insertions relative to the first copy. Consequently, they proposed that the second copy of the *COX3* gene may have lost its functionality. Understanding how duplicated non-coding regions and functional genes in cephalopod mitochondrial genomes maintain their functional roles, such as supporting replication and transcription, requires further investigation. This knowledge is expected to emerge with the accumulation of mitochondrial genome data from a broader range of cephalopod species. The Ka/Ks ratio, which compares non-synonymous to synonymous substitution rates, is commonly used to assess positive Darwinian selection and evolutionary relationships between species, both homogeneous and heterogeneous, at the molecular level [73]. A Ka/Ks ratio below 1 suggests negative or purifying selection, indicating evolutionary constraints that eliminate harmful mutations. A ratio of 1 represents neutral evolution, where mutations neither offer advantages nor disadvantages. In contrast, a ratio above 1 indicates positive or diversifying selection, pointing to adaptive changes driven by evolutionary pressures. In mollusks, species with low mobility exhibit a higher Ka/Ks ratio than those with high mobility, suggesting a greater accumulation of non-synonymous mutations in the mtDNA of less mobile species [73,74]. In this study, the Ka/Ks ratios of all 13 protein-coding genes (PCGs) in the two forms of *S. oualaniensis* were consistently below 1, indicating that these genes were under strong purifying selection to preserve their functional integrity. Among the 13 PCGs, ND4 exhibited the highest Ka/Ks ratio, a pattern similarly observed in other Oegopsida species, suggesting a relatively fast evolutionary rate and potential adaptive changes [25,66]. In contrast, *COX2* had the lowest Ka/Ks ratio, reflecting strong purifying selection and slower evolutionary rates. These findings highlight the differential selective pressures acting on mitochondrial genes, underscoring their functional importance and evolutionary dynamics.

The phylogenetic analyses yielded well-supported and congruent topologies across both Bayesian Inference (BI) and Maximum Likelihood (ML) approaches, clearly delineating three primary clades within the family Ommastrephidae, and the tree includes branching patterns supported by posterior probabilities and bootstrap value (indicated at the nodes). These clades reflect evolutionary divergence consistent with ecological, morphological, and genetic distinctions among the included genera. This primary clade includes species from the genera *Dosidicus*, *Eucleoteuthis*, *Sthenoteuthis*, and *Ommastrephes*, all belonging to the subfamily Ommastrephinae. The strong support for this grouping, as evidenced by high bootstrap values and posterior probabilities, validates its monophyletic status. These species are broadly distributed across tropical and subtropical waters and exhibit significant adaptations to pelagic lifestyles. Key traits such as rapid growth rates, high fecundity, and extensive migratory behaviors have likely driven their evolutionary success and diversification. Notably, within the Ommastrephinae clade, the placement of *S. oualaniensis* highlights its close evolutionary relationships with other genera, corroborating findings from previous molecular and morphological studies [62,75]. The second clade consists of species from the genus *Todarodes*, belonging to the subfamily Todaropsinae. The relationship between *T. pacificus* (Japanese flying squid) and the *D. gigas* + *E. luminosa* clade exhibits moderate to weak support (posterior probability = 0.939, bootstrap = 56). While these taxa are related, the genetic divergence indicated by branch length suggests that *T. pacificus* may have experienced unique selective pressures, potentially driven by its temperate and subarctic habitat in contrast to the tropical ranges of *Dosidicus* and *Eucleoteuthis*. This highlights the need for additional genomic markers or increased taxon sampling to better resolve this relationship, as it may involve processes such as incomplete lineage sorting or introgression [25,69,76]. Both phylogenetic trees position *I. argentinus* (Argentine shortfin squid) as an early-diverging lineage within the primary Ommastrephidae clade, reflecting significant genetic differentiation. Its placement underscores the impact of geographic isolation in the southern Atlantic Ocean and adaptation to colder waters on its evolutionary trajectory. This divergence aligns with the species’ distinct ecological and behavioral strategies, including cold-water spawning and dietary specialization, which have likely contributed to its unique evolutionary path [77,78]. The clear separation of these clades underscores the evolutionary bifurcation within Ommastrephidae, likely driven by historical geographic, ecological, and behavioral factors. The strong support for subfamily-level delineations reaffirms the utility of mitochondrial genome analysis for resolving phylogenetic relationships within this family. Furthermore, the inclusion of Architeuthis dux as an outgroup effectively anchored the tree, providing a broader context for interpreting lineage-specific divergence.

The phylogenetic analyses revealed that the multiple forms of *S. oualaniensis* clustered together, forming a distinct and robustly supported monophyletic clade. This grouping suggested a close evolutionary relationship and indicated that these two forms share a recent common ancestor. Notably, the genetic divergence observed between the dwarf and medium-sized forms aligns with levels typically associated with species differentiation, providing strong evidence for their separation as distinct evolutionary units. Additionally, in this study, the genetic divergence between the dwarf and medium-sized forms of *S. oualaniensis* across the complete mitochondrial genome reached 12%, which is nearing the highest congeneric distance of 13.9% previously reported for the Ommastrephinae subfamily, suggesting a level of genetic differentiation that may be indicative of cryptic speciation [75]. Additionally, the observed differences in the initiation and termination codon usage of protein-coding genes (PCGs) may reflect subtle variations in mitochondrial genome evolution between the two morphotypes of *S. oualaniensis*. In our earlier research, we examined genetic and size variations in *Sthenoteuthis* squids from the South China Sea, providing evidence for the differentiation between the dwarf and medium-sized forms as separate species. The medium-sized form exhibits a broader distribution, whereas the dwarf form is predominantly found in equatorial waters. Both genetic and morphological analyses reveal significant divergence, indicating the existence of pseudo-cryptic species. The observed mitonuclear discordance may be attributed to either hybridization or lineage sorting [32]. This level of divergence highlights potential cryptic speciation within *S. oualaniensis*, where traditional morphological or ecological distinctions are subtle or absent but genetic data reveal significant evolutionary separation. The clustering pattern further emphasizes the intricate structure of the *S. oualaniensis* species complex and suggests that these forms may represent separate adaptive responses to differing ecological or environmental conditions, such as variations in water temperature, depth, or prey availability. Further research integrating genetic, ecological, and morphological data is necessary to clarify the mechanisms driving this differentiation. For instance, studies on reproductive isolation, habitat preferences, and life history traits could provide additional evidence to support their classification as distinct species. These insights could also inform conservation strategies and fisheries management plans, ensuring sustainable utilization and conservation of the genetic diversity within the *S. oualaniensis* species complex. The findings have broader implications for taxonomy and evolutionary biology. The differentiation of these forms may reflect unique evolutionary pressures or adaptive strategies that have facilitated their survival and success in distinct niches. This distinction is also critical for effective resource management and conservation efforts, as treating the two forms as a single species could obscure their individual ecological roles and population dynamics. In addition, incorporating nuclear genome data in future research is crucial, as it would provide a more comprehensive understanding of the genetic divergence and help validate our mitochondrial findings, offering a more robust framework for interpreting the evolutionary relationships within *S. oualaniensis.*

## 5. Conclusions

The mitochondrial genome of the dwarf form of *S. oualaniensis* sheds significant light on the evolutionary dynamics within the Ommastrephidae family. The genome exhibits a highly conserved structure, displaying typical features of Oegopsid cephalopod mitogenomes, including seven duplicated genes, which are a distinctive characteristic of this group. Phylogenetic analysis confirms the close evolutionary relationship between the dwarf and medium-sized forms of *S. oualaniensis*, suggesting they share a recent common ancestor. The observed genetic divergence of 12% between these forms indicates the potential for cryptic speciation, implying that the two forms may represent distinct evolutionary units despite their morphological similarities. These findings underscore the value of mitochondrial genome analysis in resolving evolutionary relationships and speciation processes, particularly within the complex *S. oualaniensis* species complex. Further research, incorporating genetic, ecological, and morphological data, will be crucial for elucidating the mechanisms driving their divergence and for informing conservation strategies that ensure the sustainable management of their genetic diversity and ecological roles.

## Figures and Tables

**Table 1:** Characteristic constituents of the mitochondrial genome of two forms of *S. oualaniensis*.

	Dwarf Form of *S. oualaniensis*				Medium-Sized Form of *S. oualaniensis*			
Feature	Strand	Position	Length	Initiation/ Stop Codon	Strand	Position	Length	Initiation/ Stop Codon
*COX3*	H	1-780	780	ATG/TAA	H	1-780	780	ATG/TAA
tRNA-Ala	H	814-881	68		H	811-878	68	
tRNA-Asn	H	890-958	69		H	888-956	69	
tRNA-Ile	H	961-1026	66		H	959-1023	65	
*ND3*	H	1027-1380	354	ATG/TAA	H	1024-1377	354	ATG/TAA
*COX1*	H	1434-2966	1533	ATG/TAA	H	1421-2953	1533	ATG/TAG
*COX2*	H	2968-3655	688	ATG/AAT	H	2955-3642	688	ATG/TAA
tRNA-Asp	H	3656-3722	67		H	3643-3709	67	
*ATP8*	H	3724-3879	156	ATG/TAA	H	3711-3866	156	ATG/TAA
*ATP6*	H	3881-4573	693	ATG/TAG	H	3868-4560	693	ATG/TAG
tRNA-Phe	L	4666-4600	67		L	4652-4587	66	
tRNA-Val	L	4733-4665	69		L	4719-4651	69	
*12S rRNA*	L	5723-4734	990		L	5704-4720	985	
tRNA-Cys	L	5788-5724	65		L	5770-5705	66	
tRNA-Gln	L	5858-5792	67		L	5839-5773	67	
long non-coding region	H	5859-6419	561		H	5843-6403	561	
*COX3*	H	6420-7199	780	ATG/TAA	H	6404-7183	780	ATG/TAA
tRNA-Lys	H	7206-7273	68		H	7189-7256	68	
tRNA-Arg	H	7274-7340	67		H	7257-7323	67	
tRNA-Ser	H	7381-7448	68		H	7365-7432	68	
*ND2*	H	7449-8489	1041	ATG/TAA	H	7433-8473	1041	ATT/TAA
*COX1*	H	8461-9993	1533	ATG/TAA	H	8445-9977	1533	ATG/TAG
*COX2*	H	9995-10682	688	ATG/AAT	H	9979-10666	688	ATG/TAA
tRNA-Asp	H	10683-10749	67		H	10667-10733	67	
*ATP8*	H	10751-10906	156	ATG/TAA	H	10735-10890	156	ATG/TAA
*ATP6*	H	10908-11600	693	ATG/TAG	H	10892-11584	693	ATG/TAG
*ND5*	L	13322-11625	1698	ATG/TAA	L	13306-11609	1698	ATT/TAA
tRNA-His	L	13390-13323	68		L	13373-13307	67	
*ND4*	L	14750-13391	1360	ATA/TAG	L	14734-13374	1361	ATA/TAG
*ND4L*	L	15043-14747	297	ATG/TAG	L	15027-14731	297	ATG/TAG
tRNA-Thr	L	15116-15052	65		L	15036-15101	66	
tRNA-Ser	L	15182-15118	65		L	15167-15103	65	
*Cytb*	L	16326-15183	1144	ATA/TAG	L	16311-15168	1144	ATA/TAG
*ND6*	L	16829-16323	507	ATG/TAG	L	16814-16308	507	ATG/TAG
tRNA-Pro	L	16897-16831	67		L	16882-16816	67	
*ND1*	L	17833-16898	936	ATG/TAA	L	17818-16883	936	ATA/TAG
tRNA-Leu	L	17903-17834	70		L	17888-17819	70	
tRNA-Leu	L	17975-17909	67		L	17960-17894	67	
*16S rRNA*	L	19400-17976	1425		L	19391-17961	1431	
tRNA-Met	L	19471-19401	71		L	19462-19392	71	
tRNA-Tyr	L	19541-19476	66		L	19531-19467	65	
tRNA-Trp	L	19608-19542	67		L	19598-19532	67	
tRNA-Gly	L	19681-19617	65		L	19668-19604	65	
tRNA-Glu	L	19758-19685	74		L	19744-19672	73	
long non-coding region	H	19759-20320	562		H	19747-20308	562	

H and L refer to the heavy and light strand, respectively.

## Data Availability

The genome sequence data supporting the findings on the dwarf form of *S. oualaniensis* are publicly accessible in GenBank (NCBI) at [https://www.ncbi.nlm.nih.gov] under accession number MW542205. The authors confirm that the data supporting the findings of this study are available at the following link: https://doi.org/10.5281/zenodo.4489989 (accessed on 2 February 2021).

## Supplementary Materials

The following supporting information can be downloaded at: https://www.mdpi.com/article/10.3390/genes16020226/s1, Table S1. PCR primers were used in this research. Table S2. The details of deposited mitochondrial genomes in Genbank used in this research.
