# Supplementary material for: Characterization of the Complete Mitochondrial Genome of Dwarf Form of Purpleback Flying Squid (Sthenoteuthis oualaniensis) and Phylogenetic Analysis of the Family Ommastrephidae
Source: Genes (Basel). 2025 Feb 15;16(2):226. doi: 10.3390/genes16020226 (PMC11855653; doi:10.3390/genes16020226)
Supplement: Supplementary file 1 [file genes-16-00226-s001.zip › table S1.pdf]

Table S1. PCR primers were used in this research.

NO.	primer code	primer sequence
1	M-F1	ACATAACCAGTGAAAAGCCA
2	M-F2	CCACCCTAACACCTTCAACG
3	M-F3	AAGTCTCATCAACTCAAACCTT
4	M-F4	ACCCCTTGACCACCTATAACT
5	M-F5	TGTCACAGGAAGCAACTTAGA
6	M-F6	CTGCCTAATAATAATTCACCTCTTATGA
7	M-F7	ACATAACCCACGAACGCAG
8	M-F8	TCTACCCTCACCAACTCTTT
9	M-F9	GCACTGATCTGCCACCTTAA
10	M-F10	ACCCATTATGCAAAAGGTACGA
11	M-R1	CTACCAGGGGAGCTTGCTT
12	M-R2	AGAGCATTCAACCTAGATGAGAAAT
13	M-R3	ACGTATCTAAGTTGCTTCCTGTG
14	M-R4	GGCCTTGTGTGCTTTATTGC
15	M-R5	GGTCGAGTGTTGAGTGTGTT
16	M-R6	GCTCTTGTAGATTTACCTGCTCC
17	M-R7	TGCTGAAGGAGAGTCTGAGC
18	M-R8	CGAGAGGACCAAGGAATGCT
19	M-R9	AGGGTCATAGTGTGTGATGA
20	M-R10	CTGGTCAAAGGTCAGTGGGT
21	M-1-1F	TAATTGGAGGATTTGGAAAC
22	M-1-1R	CCAATCTGGACTATCATAACC
23	M-1-2F	TACCCGAGCTTACTTTACTTC
24	M-1-2R	GTCGATTTTGGTGTTTTACTA
25	M-2-1F	TGACTTGACATCACAACCATA
26	M-2-1R	GAAATTATAGAGAGCATTCAACC
27	M-2-2F	CAGGCACCCAACCTATGA
28	M-2-2R	TCTAAGGTACGAGCCGAAAT
29	M-3-1F	TTGGTATTTGGGCAGGAC
30	M-3-1R	TCTTTTTGTTAGAGCATTGGTT
31	M-3-2F	CGGTCACCCAGAAGTTTATA
32	M-3-2R	TCATAAGGACAAGGTTCATGTT
33	M-5-F	CGAGTTAAGTTAATATCCCCTCT
34	M-5-R	AGAGGGCTGATGTTATTGGC
35	M-10-1F	CATCGACCCATTATGCAA
36	M-10-1R	GGAGATCCTTATAATTTATAACATC
37	M-10-2F	AATTGAAAATCTCATGTGCTC
38	M2-1S	GCGATATGTACGCATTAT
39	M2-2S	GCACCCAACCTATGATTT
40	M2-3S	ATGTCCCTCATTGCTTGT
41	M2-1A	AAAGGGTTAAGAGGGTGA

42	M2-2A	TGGTTAATCAGTGGGATG
43	M2-3A	TTGGTTTGGTTTAGAGCC
44	M3-1S	TGTAAAACGACGGCCAGTTTGGTATTTGGGCAGGAC
45	M3-1A	CAGGAAACAGCTATGACCTCTTTTTGTTAGAGCATTGGTT
46	M10-1S	TGTAAAACGACGGCCAGTCATCGACCCATTATGCAA
47	M10-1A	CAGGAAACAGCTATGACCGGAGATCCTTATAATTTATAACATC
48	M10-2S	TGTAAAACGACGGCCAGTAATTGAAAATCTCATGTGCTC
49	M2-4F	AGCTTCCACTACCCCTACTATGT
50	M2-4R	AGAGAGCATTCAACCTAGATGAGA
51	M2-5F	TGACGACAGCAATACACAAACTG
52	M2-5R	AGTGAAATGGGGATTATGTTGTTT
53	M10-4F	CTTTCACTTCGAGAACATGTAAT
54	M10-4R	GTTATCATAGACACAGAAGGAGA
55	M10-5F	ACCAAAACATCATTTCCTTTACA
56	M10-5R	CAGTTTCCAAATCCTCCAATTAT
57	M3-CF1	TTTTGGTATTTGGGCAGGAC
58	M3-CR1	TATATTCTTTTTGTTAGAGCATTG
59	M3-CF2	CGGCGATACTCAGACTACCC
60	M10-CF	TATCTCGAAGACTCCCATTT
61	M10-CR	TCCTTATGGTCACCTCCC
