# Supplementary material for: Characterization of the Complete Mitochondrial Genome of Dwarf Form of Purpleback Flying Squid (Sthenoteuthis oualaniensis) and Phylogenetic Analysis of the Family Ommastrephidae
Source: Genes (Basel). 2025 Feb 15;16(2):226. doi: 10.3390/genes16020226 (PMC11855653; doi:10.3390/genes16020226)
Supplement: Supplementary file 1 [file genes-16-00226-s001.zip › table S2.pdf]

Table S2. The details of deposited mitochondrial genomes in Genbank used in this research.

**Morphological type**	**Accession numbers**	**Collection location**	**References or collector**
Architeuthis dux	KC701744	USA, Channel Islands, California	Winkelmann et al. (2015)
Dosidicus gigas	EU068697	Monterey, California, USA	Staaf et al. (2016)
Eucleoteuthis luminosa	MW450849	Western Pacific Ocean	Pei et al. (2021)
Illex argentinus	KP336702	Western Pacific Ocean	Jiang et al. (2015)
Ommastrephes bartramii	NC_020348	Eastern Pacific Ocean	Wakabayashi and Yanagimoto (2023)
Todarodes pacificus	AB158364	Sea of Japan	Yokobori et al. (2004)
Sthenoteuthis oualaniensis (medium-size form)	MT661575	South China Sea	Xu et al. (2020)
Sthenoteuthis oualaniensis (medium-size form)	EU658923	Eastern tropical Pacific Ocean	Staaf et al. (2016)
